# Changes in acceptability, consideration, intention, and uptake of direct‐to‐consumer genetic tests in the Netherlands from 2017 to 2022

**DOI:** 10.1002/jgc4.1919

**Published:** 2024-06-03

**Authors:** Anna Roos Leerschool, Anke Wesselius, Daša Kokole, Maurice P. Zeegers

**Affiliations:** ^1^ CAPHRI School for Public Health and Primary Care Maastricht University Maastricht The Netherlands; ^2^ NUTRIM School of Nutrition and Translational Research in Metabolism Maastricht University Maastricht The Netherlands; ^3^ Department of Health Promotion, Faculty of Health, Medicine and Life Sciences Maastricht University Maastricht The Netherlands

**Keywords:** acceptability, attitudes, consideration, cross‐sectional survey study, decision making, direct‐to‐consumer genetic testing, intention

## Abstract

Although the popularity of direct‐to‐consumer genetic tests (DTC‐GT) for disease‐related purposes increased, concerns persist whether consumers make well‐informed decisions about their purchase. To better target pre‐ and post‐test information materials, this study aims to determine the characteristics of people interested in undergoing DTC‐GT. In addition, it aims to determine changes in acceptability, consideration, intention, and uptake of DTC‐GT since 2017. An online cross‐sectional survey was conducted in April 2022 with a representative sample of the Dutch adult population. Ordinal regression models and chi‐squared tests were used to determine factors associated with DTC‐GT acceptability, consideration and intention, and changes in outcomes since 2017, respectively. Of the 907 included respondents, 19.3% found DTC‐GT acceptable, 29.4% considered taking a DTC‐GT in the future, 6.2% intended to take a test within the coming year, and 0.9% had already tested. High education was associated with lower acceptability, consideration, intention, and higher awareness. Respondents with a chronic disease were less likely to find DTC‐GT acceptable. Higher consideration was associated with having a partner, adopted/stepchildren, and lower age. Compared to 2017, in 2022 more respondents found DTC‐GT totally unacceptable, while more considered testing, and fewer ruled out taking a test both in the next year and the future. Education status may play an important role in people's acceptability, consideration, intention, and awareness of disease‐related DTC‐GT in the Netherlands. Easy‐to‐understand public information materials should be promoted and guidance is needed to help with decision‐making and result interpretation. Future research should focus on the best way to provide responsible guidance.

**What is known about this topic?**

Existing knowledge indicates a rising interest in direct‐to‐consumer genetic testing (DTC‐GT) for personalized disease risk assessment, with concerns about information adequacy and potential misinterpretation of results among consumers.

**What this paper adds to the topic?**

This paper contributes by revealing a shift in public attitudes over 5 years, indicating increased consideration to undergo DTC‐GT, emphasizing the role of education in shaping perceptions, and underscoring the need for accessible and comprehensible information materials to facilitate informed decision‐making and results interpretation.

## 1 INTRODUCTION

The public's desire for greater health responsibility is rising, along with the trend toward personalized healthcare driven by Big Data (Cherkas et al., 2010; Naithani et al., 2021; Rose, 2001). This involves tailoring healthcare to individual needs, including personalized disease prevention. Direct‐to‐consumer genetic tests (DTC‐GT) aimed at estimating personal disease risks, have become increasingly popular, with over 50 companies offering such tests to Dutch consumers by April 2020 (Rigter et al., 2020).

DTC‐GT for disease purposes utilize the public's desire to take more responsibility for their own health (Cherkas et al., 2010), by offering direct access to one's genetic profile. In contrast to genetic testing in the traditional healthcare setting, DTC‐GT are of preventive nature rather than diagnostic and are sold mostly via the internet circumventing the involvement of a healthcare professional.

While this has been suggested to promote autonomy (Chung & Ng, 2016; Vayena, 2015), there is concern that the public lacks sufficient information and support to make informed choices about testing and to understand test results and their consequences (Cernat et al., 2022; Pavarini et al., 2021; Stewart et al., 2018).

The public's knowledge of DTC‐GT results and genetic concepts remains relatively low (Calabrò et al., 2020; Cernat et al., 2022; Gerdes et al., 2021), compounded by a lack of knowledge among healthcare professionals to assist consumers (Martins et al., 2022). Particularly concerning is the finding that individuals with limited understanding of DTC‐GT interpretation are more inclined to undergo testing, potentially leading to unpreparedness for received information or misinterpretation of results (Cernat et al., 2022; Stewart et al., 2018; Wasson et al., 2013).

Interpretation of DTC‐GT results is a major challenge faced by consumers. Misinterpretation of results could lead to unwarranted stress, wrong health decisions, and unnecessary tests and visits to healthcare providers (Cernat et al., 2022; Rafiq et al., 2015). Involvement of trained genetic counselors is increasing, but consumers typically must decide whether to seek this option independently (Harris et al., 2013). With limited reliable pre‐ and post‐test resources available, many consumers turn to online communities or third‐party interpretation sites to supplement their understanding of genetic test reports (Mukherjee et al., 2022; Nelson et al., 2019).

Considering the above points and that undergoing DTC‐GT can be a vulnerable moment in a person's life (Pavarini et al., 2021), there is a need for support to aid individuals in making informed choices and interpreting their results accurately. Genetic counselors, with their specialized training in genetics and counseling, are uniquely positioned to provide this support (Blout Zawatsky et al., 2023). An assessment of the people currently interested in testing could help tailor such pre‐ and post‐test support. Therefore, the aim of this study is to identify characteristics of people who (1) find DTC‐GT for disease purposes acceptable, (2) consider undergoing a disease‐related DTC‐GT in the distant (consideration) or near (intention) future, and (3) have taken such a DTC‐GT.

A survey study with a similar aim was conducted by our research team in 2017 (Stewart et al., 2018). We anticipate an increase in the number of respondents who have undergone genetic testing since then, as well as a greater prevalence of formed opinions regarding DTC‐GT. With more information available, uncertainty toward DTC‐GT may have decreased, allowing individuals to form opinions (Rogers, 2003). Therefore, another aim is to determine how acceptability, consideration, intention of uptake, and actual uptake of disease‐related DTC‐GT have changed in the Dutch adult population compared to the last survey conducted by our research team in 2017.

## 2 METHODS

### 2.1 Design

This cross‐sectional analysis of disease‐related DTC‐GT is part of a study into the acceptability, consideration, intention, and uptake of six different types of DTC‐GT. It is also a partial update of and comparison to the work conducted in 2017 by Stewart et al. (2018). Items included in the current questionnaire were kept as they were asked in the 2017 survey, except for small changes to the questions on awareness, chronic disease diagnosis, having a family member diagnosed with a genetic disease, and self‐rated health, as mentioned below. The questionnaire was pretested by the internet research company *Flycatcher* (Maastricht, The Netherlands) in a small group of respondents with similar characteristics to the target audience (Flycatcher Internet Research, 2024). The survey was voluntary, confidential, and anonymous, and complies with the General Data Protection Regulation (GDPR). The study was approved by the Ethics Review Committee of Maastricht University (FHML‐REC/2021/085).

### 2.2 Participants

Participants were recruited from Flycatcher's online panel representative of the Dutch adult population (18+), based on age, gender, education, and province. The representativeness of the panel is determined using the most recent version of the so‐called “Gold Standard”, a calibration tool specially developed by the Center for Marketing Insights, Research & Analytics (MOA) in collaboration with Statistics Netherlands (CBS) (Data and Insights Network, 2024). Flycatcher is in the possession of the quality label for market, opinion, and social research (ISO 20252) ensuring that Flycatcher's research activities, including the Flycatcher panel, meet the international organization for standardization (ISO) quality requirements with regard to, among other things, research confidentiality, competencies and training of employees, transparency and guidelines for all aspects of a research process (International Organization for Standardization, 2019).

The panel consists of more than 10,000 adults who have voluntarily and actively agreed to participate in online surveys via ‘double‐active‐opt‐in’. Panel members cannot select the type of surveys for which they wish to be invited. Flycatcher panelists receive a predetermined number of points and one ticket in the Flycatcher Quarterly Lottery for each fully completed questionnaire. Points can be redeemed for a gift certificate. A personalized link to the questionnaire was sent to each participant via email. To prevent persons other than the person for whom the questionnaire was intended from filling in the survey, the first page included an authentication question. This was followed by informed consent and a participant information letter. All persons gave their informed consent prior to their inclusion in the study.

### 2.3 Data collection

The survey started with a description of DTC‐GT. Links to short videos and webpages on the different types of DTC‐GT (including for disease‐related tests) were included, to give participants some background on the common genetic tests available (Text S1). After this introduction to DTC‐GT, a 15‐min questionnaire started. The results of this report focus on DTC disease‐related tests which we defined to include both (1) DTC genetic disease‐risk testing (aimed at estimating an individual's risk of developing chronic diseases in the future) and (2) carrier status testing (to test whether an individual carries a single copy of a genetic pathogenic variant which will cause a disorder when present in both copies).

The questionnaire was sent out on March 25th, and could be completed until April 4th, 2022. To increase the response rate, on March 29th a reminder e‐mail was sent to all panelists who had not yet started the questionnaire or had not completed it by that time. An additional group was invited on March 31st. Non‐respondents were considered to be people who did not click on the survey link in the mail, did not give consent, or did not complete the entire survey, as well as those who did not complete the survey seriously according to Flycatcher's quality control. To maximize the quality of responses, participants could go back to change answers and all questions had to be completed to be able to submit the questionnaire.

At first, participants were asked whether they had ever purchased a DNA test to receive disease‐related information of their personal genetic profile. This was followed by questions on the following quantitative items:

#### 2.3.1 Awareness

While in 2017, awareness of DTC‐GT for disease‐related purposes was asked using a single question (yes/no answer), in 2022 the question was split into two parts. Respondents were first asked “Prior to participating in this research, had you ever heard of DTC‐GT?” (yes/no answer). Subsequently, only respondents who answered “yes” were asked “Of which type of DTC‐GT had you heard before?”. Respondents could select either of the six different types of DTC‐GT, including disease‐related DTC‐GT. Multiple answers were accepted.

#### 2.3.2 Outcome parameters

Acceptability of DTC‐GT for disease‐related purposes was measured as “How acceptable is it in your opinion that private companies provide results of disease‐related genetic tests directly to consumers, without mandatory involvement of a consultant (doctor, geneticist, coach or counselor)?” This was rated on a 5‐point scale (1—Completely unacceptable to 5—Completely acceptable).

Consideration of undergoing DTC‐GT for disease‐related purposes at some time in the future was measured using the following question: “Would you consider doing a DTC‐GT for disease‐related purposes at some point in the future?” This question aimed to determine the degree of willingness to think about undergoing testing, but not yet intending to do so.

The outcome intention was defined as intention to undertake a disease‐related DTC‐GT in the coming year. Intention was measured using the question: “Do you intend to use a DTC‐GT for disease‐related purposes in the next year?” Compared to consideration, intention reflects a more committing intent to undergo testing.

Both consideration and intention were rated on a 5‐point scale (1—Certainly no to 5—Certainly yes).

#### 2.3.3 Independent variables

The subsequent independent variables were kept the same as in Stewart et al. (2018): age, gender, education (less/middle/high), partner (yes/no), planning to have children (yes/maybe/no/don't know, where “no” refers to people with children who are not planning to have more, and people with no children who never plan to have them), having biological children (yes/no), having adopted or stepchildren (yes/no). Less education included none or primary school education, pre‐vocational education, and junior secondary school education. Middle education comprised vocational training, senior secondary school education, and completion of the first year of university or HBO (hoger beroepsonderwijs; higher professional education). High education encompassed completed university‐level education or HBO programs. The answer option “I would rather not say” was added to the independent variables: chronic disease diagnosis (yes/no/don't know/I would rather not say), having a family member diagnosed with a genetic disease (yes/no/don't know/I would rather not say), and self‐rated health (1—Poor to 5—Excellent/ I would rather not say). Moreover, respondents were asked additional permission prior to answering a question about their religion (yes/no), in accordance with the GDPR.

### 2.4 Data analysis

At first, univariable ordinal regression analyses were performed to identify variables associated with acceptability, consideration, and intention of DTC‐GT. Factors that met the cutoff of *α* < 0.20 in the univariable analyses were selected to be further tested in multivariable ordinal regression models. Binary logistic regression was used to test the association between education status and awareness of disease‐related DTC‐GT. Goodness of fit of the models was determined using the Pearson chi‐squared statistic in SPSS. To test the assumption of proportional odds, a cutoff value of *p* < 0.01 was taken to indicate a statistically significant result on the test of parallel lines. Linearity was checked between the continuous variables age and self‐rated health and each outcome using the Box‐Tidwell transformation.

To determine changes in the frequencies of the outcomes between 2017 and 2022, the chi‐squared statistic was used.

SPSS was used for all analyses and a *p*‐value of <0.05 was considered statistically significant.

## 3 RESULTS

### 3.1 Study population

Of the 1964 panelists who were contacted, 1265 responded to the questionnaire (64% response rate). Of these, 10 were excluded due to low response quality and 348 did not consent to participate, dropped out, or did not complete the questionnaire. Therefore, 907 respondents with complete data were included in our analyses.

Table 1 lists the main characteristics of the study sample. The population was comparable to the Dutch population in terms of age, gender, and education. More respondents had completed a middle level of education (42.8%) than less (24.4%) or high (32.9%) education, in line with the distribution in the Dutch population. Most respondents had a partner (71.0%) and were non‐religious (62.4%). Around three quarters (73.9%) of respondents were not planning to have children, and more than half (58.9%) already had biological children. Fewer respondents had adopted or stepchildren (11.1%). Almost half (49.9%) of respondents rated their health as “good”, around a fifth as “very good” (21.4%) and “fair” (19.5%), and much fewer as excellent (5.2%) or poor (3.9%). Over a third (35.2%) reported having a chronic disease and 22.3% had a family member with a genetic disease. Non‐respondents did not differ from respondents by gender or age, but a greater proportion of non‐respondents was seen in the less education category and a smaller proportion in the high education category (*χ* ^2^ (2, N = 1971) = 6.65, *p* = 0.036).

**TABLE 1:** Descriptive statistics of the 2022 and 2017 study samples.

	2022			2017		
	*n*	%	Dutch general population %^a^ ^,^ ^b^	*n*	%	Dutch general population %^b^ ^,^ ^c^
Total	907			836		
Age						
18–39	302	33.3	35	250	29.9	35
40–59	318	35.1	34	299	35.8	38
60+	287	31.6	32	287	34.3	30
Gender						
Male	463	51.0	49	422	50.5	49
Female	444	49.0	51	414	49.5	51
Education level						
Less	221	24.4	27	272	32.5	31
Middle	388	42.8	43	362	43.3	44
High	298	32.9	31	202	24.2	25
Partner						
Yes	644	71.0		600	71.8	
No	263	29.0		236	28.2	
Religious						
Yes	313	34.5		357	42.7	
No	566	62.4		479	57.3	
Missing	28	3.1		NA	NA	
Planning to have children						
Yes	159	17.5		129	15.4	
Maybe	43	4.7		29	3.5	
No	670	73.9		640	76.6	
Don't know	35	3.9		38	4.5	
Biological children						
Yes	534	58.9		518	62.0	
No	373	41.1		318	38.0	
Adopted or stepchildren						
Yes	101	11.1		57	6.8	
No	806	88.9		779	93.2	
Self‐rated health						
Excellent	47	5.2		46	5.5	
Very good	194	21.4		144	17.2	
Good	453	49.9		431	51.6	
Fair	177	19.5		187	22.4	
Poor	35	3.9		28	3.3	
I would rather not say	1	0.1		NA	NA	
Having a chronic disease						
Yes	319	35.2		303	36.2	
No	555	61.2		533	63.8	
I would rather not say/don't know	33	3.6		NA	NA	
Genetic disease in the family						
Yes	202	22.3		190	22.7	
No	523	57.7		450	53.8	
I would rather not say/don't know	182	20.1		196	23.4	
Previous use of DTC‐GT for disease‐related purposes						
Yes	8	0.9		2	0.2	
No	899	99.1		834	99.8	
Awareness of DTC‐GT for disease‐related purposes						
Yes	179	19.7		238	28.5	
No	728	80.3		598	71.5	

^a^ “Gold Standard” – Statistics Netherlands (CBS) in collaboration with the Center for Marketing Insights, Research & Analytics (MOA) 2021.

^b^ Percentages may not add up to 100% due to rounding.

^c^ “Gold Standard” – CBS in collaboration with MOA 2016.

**FIGURE 1:**
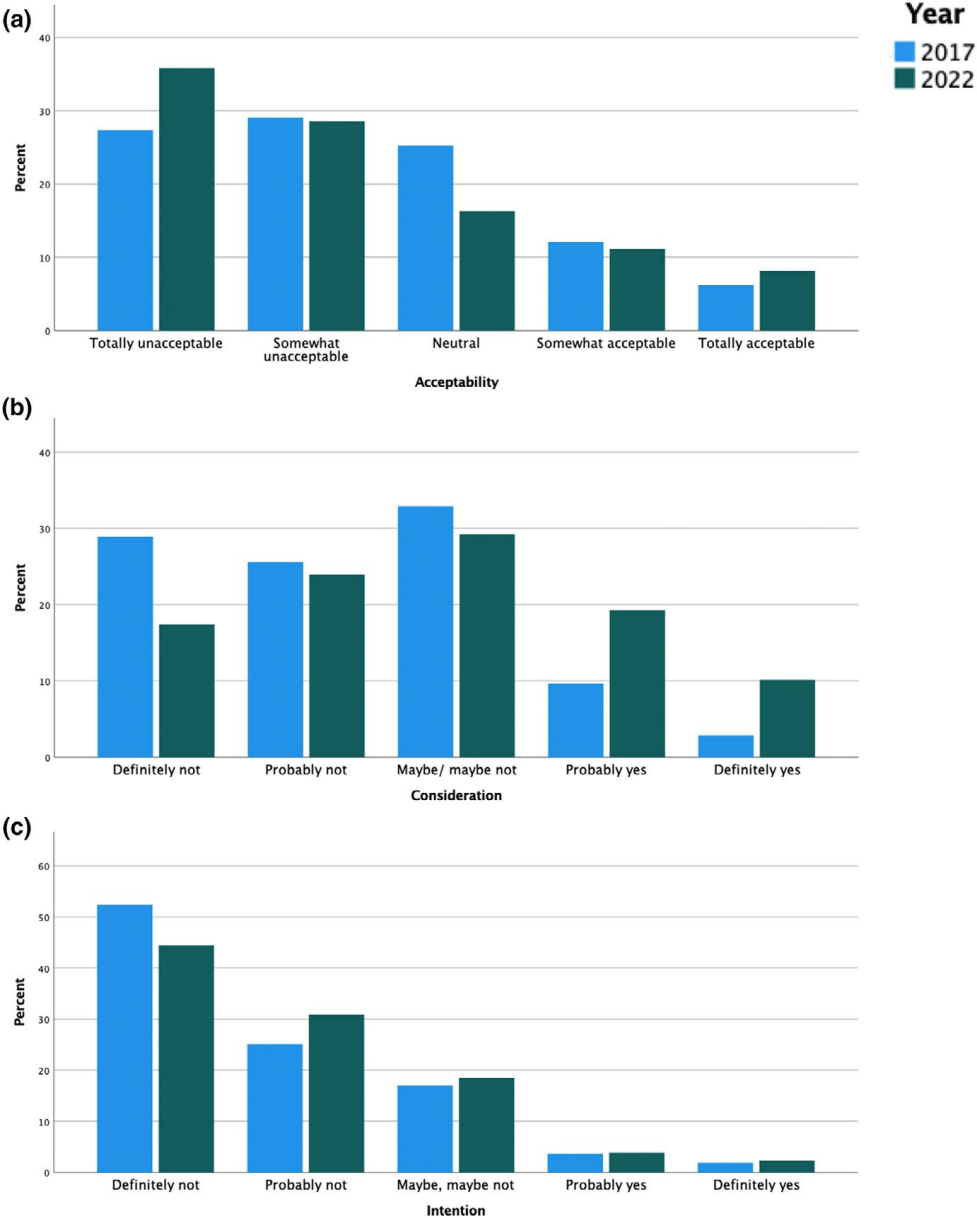
Showing (a) acceptability, (b) consideration, and (c) intention of uptake of disease‐related DTC‐GT in 2017 and 2022.

Almost a fifth (19.3%) of the respondents in our survey found DTC‐GT somewhat or totally acceptable. Nearly a third (29.4%) probably or definitely considered taking a DTC‐GT at some point in the future, while much fewer (6.2%) respondents probably or definitely intended to take a test in the coming year (Table 2, Figure 1). Around 1 in 100 people (0.9%) had already taken a test (Table 2).

**TABLE 2:** Cross‐sectional comparison of acceptability, consideration, intention, and actual uptake of disease‐related DTC‐GT, 2017 versus 2022.

	2017	2022	Chi‐squared test	
	*n* = 836	*n* = 907		
	*n* (%)	*n* (%)		
Acceptability				
Totally unacceptable	229 (27.4)	325 (35.8)	14.29	*p* < 0.001*
Somewhat unacceptable	243 (29.1)	259 (28.6)	0.55	*p* = 0.814
Neutral	211 (25.2)	148 (16.3)	21.17	*p* < 0.001*
Somewhat acceptable	101 (12.1)	101 (11.1)	0.38	*p* = 0.538
Totally acceptable	52 (6.2)	74 (8.2)	2.44	*p* = 0.118
*χ* ^2^ = 29.20, *p* < 0.001*				
Consideration				
Definitely not	242 (28.9)	158 (17.4)	32.69	*p* < 0.001*
Probably not	214 (25.6)	217 (23.9)	0.65	*p* = 0.419
Maybe/maybe not	275 (32.9)	265 (29.2)	2.75	*p* = 0.097
Probably yes	81 (9.7)	175 (19.3)	32.03	*p* < 0.001*
Definitely yes	24 (2.9)	92 (10.1)	37.04	*p* < 0.001*
*χ* ^2^ = 89.48, *p* < 0.001*				
Intention				
Definitely not*	438 (52.4)	403 (44.4)	11.04	*p* < 0.001*
Probably not*	210 (25.1)	280 (30.9)	7.12	*p* = 0.008*
Maybe/maybe not	142 (17.0)	168 (18.5)	0.70	*p* = 0.402
Probably yes	30 (3.6)	35 (3.9)	0.89	*p* = 0.766
Definitely yes	16 (1.9)	21 (2.3)	0.34	*p* = 0.561
*χ* ^2^ = 11.83, *p* = 0.019*				
Uptake				
Yes	2 (0.2)	8 (0.9)	3.15	*p* = 0.076

*Note*: Chi‐squared test *n* = 1743.

* *p* < 0.05.

### 3.2 Multivariable analyses

There were no statistically significant results (*p* < 0.01) for the test of parallel lines, indicating that the proportionality assumption was met for all analyses. The linearity test for acceptability and age in years was the only test which showed that linearity was violated (*p* = 0.004; Table S1). Therefore, age was only entered as a categorical variable into the univariable and multivariable analyses for acceptability, and self‐rated health was entered as a continuous variable in all cases (Table 3, Table S1). Twenty‐eight respondents (1.6%) did not give permission to answer a question about religion. Multivariable analyses with and without religion gave the same conclusions.

**TABLE 3:** Multivariable analyses for acceptability, consideration, and intention of DTC‐GT for disease‐related purposes.

	*b*	SE *b*	*p*‐value
Acceptability			
Gender			
Female	−0.156	0.128	0.223
Male	Ref		
Age			
18–24	Ref		
25–34	0.184	0.263	0.484
35–44	0.263	0.304	0.387
45–54	0.102	0.325	0.753
55–64	0.032	0.332	0.922
65–74	−0.085	0.336	0.801
75+	−0.726	0.387	0.061
Education			
Less	Ref		
Medium	−0.174	0.158	0.271
High	−0.663	0.175	<0.001*
Having a partner			
Yes	0.228	0.142	0.108
No	Ref		
Planning to have children			
Yes	−0.143	0.253	0.572
Maybe	0.446	0.314	0.156
Don't know	0.842	0.354	0.017*
No	Ref		
Having a chronic disease			
Yes	−0.312	0.132	0.019*
I would rather not say/don't know	−0.454	0.336	0.176
No	Ref		
Consideration			
Age in years			
Per 1‐year increase	−0.025	0.005	<0.001*
Education			
Less	Ref		
Medium	0.133	0.158	0.401
High	−0.417	0.173	0.016*
Having a partner			
Yes	0.307	0.147	0.037*
No	Ref		
Being religious			
Yes	−0.119	0.129	0.357
No	Ref		
Planning to have children			
Yes	−0.006	0.214	0.977
Maybe	0.592	0.311	0.057
Don't know	0.231	0.342	0.500
No	Ref		
Having biological children			
Yes	0.051	0.151	0.734
No	Ref		
Having adopted or stepchildren			
Yes	0.486	0.197	0.014*
No	Ref		
Genetic disease in the family			
Yes	0.059	0.150	0.694
I would rather not say/don't know	0.146	0.158	0.355
No	Ref		
Intention			
Education			
Less	Ref		
Medium	−0.071	0.157	0.652
High	−0.341	0.174	0.050*
Planning to have children			
Yes	0.213	0.169	0.207
Maybe	0.561	0.288	0.051
Don't know	0.079	0.328	0.810
No	Ref		
Self‐rated health			
Per 1 point increase in score	−0.098	0.074	0.184

* *p* < 0.05.

Less and middle education were associated with lower awareness of DTC‐GT compared to high education (less vs. high education *b* = −0.979, *p* = <0.001; middle vs. high education *b* = −0.597, *p* = 0.001).

#### 3.2.1 Acceptability

More highly educated respondents were less likely to find DTC‐GT acceptable compared to respondents with less education (*b* = −0.663, *p* = <0.001). Similarly, having a chronic disease was associated with lower acceptability (*b* = −0.312, *p* = 0.019). Individuals who answered don't know to whether they wanted to have children, were more acceptable of DTC‐GT than people who were not planning to have children (*b* = 0.842, *p* = 0.017; Table 3).

#### 3.2.2 Consideration and intention

Consideration and intention were lower amongst respondents who have a high education, compared to those with less education (consideration *b* = −0.459, *p* = 0.009; intention *b* = −0.341, *p* = 0.050). Further, there was greater consideration amongst younger respondents (per 1 year increase in age *b* = −0.025, *p* = <0.001), those with a partner (*b* = 0.328, *p* = 0.026), and those with adopted or stepchildren (*b* = 0.495, *p* = 0.012), but not amongst people with biological children (*b* = 0.064, *p* = 0.669; Table 3). In contrast, greater intention was not associated with age (*b* = −0.003, *p* = 0.456), having a partner (*b* = 0.031, *p* = 0.820), nor with having adopted or stepchildren (*b* = 0.190, *p* = 0.327) in the univariable analyses (Table S1).

### 3.3 Changes in descriptive statistics between 2017 and 2022

There were more respondents with a high education in 2022 (32.9%) than in 2017 (24.2%) and fewer respondents with less education in 2022 (24.4%) than in 2017 (32.5%) (*χ* ^2^ (2, *N* = 1743) = 21.75, *p* < 0.001). Furthermore, less people indicated that they were religious in 2022 (35.6%) vs. 2017 (42.7%) (*χ* ^2^ (1, *N* = 1715) = 9.06, *p* = 0.003) and more respondents had adopted or stepchildren (2022: 11.1%, 2017: 6.8%) (*χ* ^2^ (1, *N* = 1743) = 9.84, *p* = 0.002). Awareness of DTC‐GT for disease‐related purposes was lower in 2022 (19.7%) than in 2017 (28.5%) (*χ* ^2^ (1, *N* = 1743) = 18.23, *p* < 0.001; Table 1).

### 3.4 Changes in acceptability, consideration, intention, and uptake between 2017 and 2022

Table 2 compares the findings for all outcomes in 2017 with 2022. There were statistically significant differences in acceptability, consideration, and intention between 2022 and 2017, but not in actual uptake. In 2022, 35.8% of the respondents found DTC‐GT for disease‐related purposes totally unacceptable compared to 27.4% in 2017 (*χ* ^2^ (1, *N* = 1743) = 14.29, *p* < 0.001). Moreover, there were fewer people with a neutral opinion on DTC‐GT in 2022 (16.3%) than in 2017 (25.2%) (*χ* ^2^ (1, *N* = 1743) = 21.17, *p* < 0.001). More people probably (*χ* ^2^ (1, *N* = 1743) = 32.03, *p* < 0.001) or definitely (*χ* ^2^ (1, *N* = 1743) = 37.04, *p* < 0.001) considered undertaking a DTC‐GT in 2022 (19.3% and 10%, respectively) than in 2017 (9.7% and 2.9%, respectively), and in 2022 versus 2017 fewer respondents ruled out ever taking a test in the future (17.4% vs. 28.9%; *χ* ^2^ (1, *N* = 1743) = 32.69, *p* < 0.001) or within the next year (44.4% vs. 52.4%; *χ* ^2^ (1, *N* = 1743) = 11.04, *p* < 0.001; Table 2, Figure 1).

## 4 DISCUSSION

Nearly a fifth of the respondents in our survey found DTC‐GT somewhat or totally acceptable, and a third considered taking a DTC‐GT at some point in the future, although much fewer (6.2%) respondents intended to take a test in the coming year. Around 1 in 100 people (0.9%) had already taken a test.

### 4.1 Awareness

Awareness of DTC‐GT for disease‐related purposes was statistically significantly lower in 2022 (19.7%) than in 2017 (28.5%). This was an unexpected result, especially considering the current worldwide exponential growth in consumer genetic test uptake (Janzen, 2014; Khan & Mittelman, 2018). However, this finding may be related to reduced marketing of disease‐related DTC‐GT in the Netherlands since 2017, although, to our knowledge, no research exists to support this. Further, there was a slight difference in the way the awareness questions were phrased between the years, which may have contributed to the difference. As a result of splitting the question into two parts, only respondents who answered “yes” to the question on overall DTC‐GT awareness were asked about which types of DTC‐GT they had heard of before. This led to fewer people being asked the question specifically about the different types of DTC‐GT, including tests for disease‐related purposes. Moreover, while multiple answer options were accepted, it is possible that the popularity of ancestry testing overshadowed disease‐related testing.

### 4.2 Acceptability

The majority of respondents (64.4%) found DTC‐GT for disease purposes unacceptable. This is an 8.4% increase from 2017. This may be partially attributed to the differences between the populations in 2022 and 2017, with there being more highly educated people in 2022. Higher education was associated with lower acceptability in the current survey. Further possible reasons for people's low acceptability could be due to growing concerns about privacy, test validity and utility, companies' lack of providing genetic expertise and counseling, and worries that results may cause distress and anxiety (Critchley et al., 2015; Hui et al., 2021; Mavroidopoulou et al., 2015). The latter was found to be the main reason people abstained from DTC‐GT in a review of six European studies (Hoxhaj et al., 2020). Lastly, the phrasing of the question on acceptability may have been interpreted as though involvement of a consultant is mandatory and that therefore, taking a DTC‐GT without the involvement of a consultant is less acceptable. On the other hand, there was also a slight increase in respondents who found DTC‐GT acceptable (although not statistically significant) and fewer respondents were “neutral” (8.9% decrease). Taken together, these findings reflect assumptions regarding the diffusion of innovations that more people have formed an opinion about DTC‐GT over time (Rogers, 2003).

### 4.3 Consideration, intention, and uptake

Consideration of DTC‐GT for disease purposes has significantly increased over the years among the Dutch population. While in 2022 almost a third of the population considered testing, in 2017 this was just 12.6%, (Stewart et al., 2018) and in 2010 a mere 6% expressed interest in DTC‐GT for a genetic predisposition to specific chronic diseases (Vermeulen et al., 2014). These findings could reflect that with increasing popularity of DTC‐GT, more people are thinking about testing. Alternatively, this could reflect the public's increased sense of responsibility for one's own health.

Our finding of consideration being higher than acceptability is somewhat surprising. In contrast to the question on acceptability, the questions regarding consideration and intention do not explicitly mention the involvement of a consultant. As mentioned earlier, this may have contributed to lower acceptability. However, all three questions were identical to those in the 2017 survey, in which consideration was not found to be greater than acceptability. Therefore, this interesting finding may reflect that respondents in 2022 are more interested in DTC‐GT than in 2017, but do not find testing acceptable unless a consultant is present. With public concerns about DTC‐GT growing, the importance of adequate support surrounding DTC‐GT is becoming more apparent (Critchley et al., 2015; Hui et al., 2021; Mavroidopoulou et al., 2015).

Similar to consideration, intention and uptake have also increased since 2017 but these findings are not statistically significant. Notably, for uptake, this is likely due to few actual uptake cases in both years, resulting in low statistical power. Our findings show that both intention to test and actual uptake are still low in the Netherlands. This is in line with recent research on DTC‐GT uptake in the Netherlands (Bos et al., 2022). Similarly, previous studies conducted in other countries show little evidence of significant uptake of DTC‐GT for disease‐related purposes, albeit fluctuations between geographical regions (ranging from <1% in Denmark to 3.8%–11% in the US) (Carroll et al., 2020; Gerdes et al., 2021; Hui et al., 2021; Metcalfe et al., 2019; Ruhl et al., 2019; Tiner et al., 2022). This aligns with the findings of Charbonneau et al. (2020) that intention of DTC‐GT was the highest for the US, followed by Australian, UK, and Japanese respondents. Given the dominance of the US market, this is unsurprising.

The differences in this survey between intention and actual uptake reflect the common discrepancy between people's intentions and their actual behavior, known as the intention behavior gap. “Inclined abstainers”, people who intend to change their behavior but do not, have been shown to be mainly responsible for the intention‐behavior gap (Godin & Conner, 2008; Orbell & Sheeran, 1998; Rhodes & De Bruijn, 2013; Sheeran, 2002). Current research suggests that intentions get realized around 50% of the time overall, with an even lower rate of 30–40% for health behaviors (Armitage & Conner, 2001; Rhodes & De Bruijn, 2013; Sheeran & Webb, 2016). This corresponds somewhat with our findings, as actual uptake (0.9%) is approximately 40% of the 2.3% of people who definitely intend to take a test. However, due to the cross‐sectional nature of our study, this finding of uptake reflects previous uptake and cannot inform how many people will actually go on to purchase a test. As outlined by the Theory of Planned Behavior (Ajzen, 1991), most behaviors to some extent depend on non‐motivational factors such as money and time, and in the case of purchasing a DTC‐GT, possibly internet access/familiarity with using the internet (Sanderson et al., 2010). While there is a vast amount of research on moderators of the intention‐behavior gap for health behaviors, research for genetic testing specifically is limited. One such example is a study by Sanderson et al. (2010) who examined factors associated with interest in and uptake of genetic testing for lung cancer risk in smoking relatives of lung cancer patients. While they reported a modest but significant relationship between interest and uptake, their findings also suggest that factors associated with interest in genetic testing differ from correlates of uptake (Sanderson et al., 2010). Future prospective studies are warranted to gain a more comprehensive understanding of the uptake of DTC‐GT in the Netherlands and to determine associated factors.

### 4.4 Factors associated with acceptability, consideration, and intention

In the current survey, high education was associated with lower acceptability, consideration, and intention. Respondents with a chronic disease were less likely to find DTC‐GT acceptable and higher consideration was statistically significantly associated with having a partner, adopted/stepchildren, and lower age. In contrast to previous research, we found no association with self‐rated health (Roberts et al., 2017; Stewart et al., 2018), religion (Mählmann et al., 2016; Stewart et al., 2018), gender (Bloss et al., 2010; Cherkas et al., 2010; Dong et al., 2022; Hall et al., 2012; Roberts et al., 2017), or having a genetic disease in the family (Mählmann et al., 2016; Stewart et al., 2018). Notably, there was no overlap between the associated factors in 2017 and 2022, possibly due to the differences between the populations.

Our results suggest that particularly education may play an important role in contributing to people's current decisions about testing in the Netherlands. While no association was found between education status and interest in DTC‐GT in a recent study in Hong Kong (Hui et al., 2021), the current findings resonate with other research in that people's perceptions of DTC‐GT vary by level of education (Hall et al., 2012; Vermeulen et al., 2014).

The negative associations between education and acceptability, consideration, and intention could be explained by higher educated respondents having more knowledge about genetics (Morren et al., 2007; Vermeulen et al., 2014). In turn, respondents with more knowledge about testing have been shown to find DTC‐GT for disease susceptibility less acceptable (Stewart et al., 2018). As previously pointed out by Stewart et al. (2018), this may suggest that providing the public with improved information provision could lower interest in taking a test, and hence, possibly actual uptake. Moreover, higher education has previously been associated with increased concerns related to DTC testing (Bloss et al., 2010; Mavroidopoulou et al., 2015). On the contrary, however, more knowledge about genetics has also been associated with a more favorable attitude toward genetic testing in general (Morren et al., 2007; Vermeulen et al., 2014).

While further research is carried out to clarify the association, it is paramount that DTC‐GT information materials are comprehensible to people of all educational backgrounds. Although the education level in the Netherlands is relatively high, more than a quarter of the population has less education. Our results suggest that individuals with less education may be more interested in, yet less aware of disease‐related DTC‐GT. Together with research showing that less education is associated with less knowledge about genetics (Morren et al., 2007; Vermeulen et al., 2014), this indicates that an informed decision about testing may often not be made. This suggests the need for the dissemination of easy‐to‐understand information materials and guidance for the public, particularly in their decision‐making, but also with interpretation of results to limit the risks of DTC‐GT for disease purposes. On a related note, a recent Dutch study found a public need for more pre‐ and post‐test support to help people understand what to expect from testing, interpret results, and, where relevant, adopt a healthier lifestyle. This includes the need for transparent information on how test results are calculated and how data are stored (Bemelmans et al., 2023).

Previous studies and professional organizations call for more involvement of independent genetic counselors to best guide people considering DTC‐GT (Blout Zawatsky et al., 2023; Hudson et al., 2007; National Society of Genetic Counselors, 2019). While most genetic counselors agree that this would improve DTC‐GT (Hsieh et al., 2021), one‐on‐one counseling may be unrealistic, especially given the typical diagnostic setting of genetic counseling in the Netherlands, and the limitations faced by genetic counselors, such as time constraints and potential lack of confidence in counseling around DTC‐GT results (Hsieh et al., 2021; Miura et al., 2023). However, genetic counselors could offer important and unique expertise in the development of (online) information resources around DTC‐GT, such as a decision aid (Rigter et al., 2020). The results from this study can help tailor such resources to those most interested in testing. In addition, genetic counselors could help in educating other healthcare providers, such as general practitioners, who are currently the recommended first line of support for people with questions surrounding DTC‐GT in the Netherlands (Rijksinstituut voor Volksgezondheid en Milieu, 2024).

### 4.5 Strengths and limitations

This study was performed on a large sample of 907 respondents representative of the Dutch population in 2022. There are, however, some limitations to this research. (1) In order to compare the findings to our previous survey, a similar questionnaire was used as in 2017. This included, where possible, validated or previously used questionnaires, but also several self‐developed items. As much as possible, existing questionnaires were modified to fit the research, to improve the validity of the questionnaire. The questionnaire was also successfully pretested in a small sample. (2) Furthermore, most respondents (80.3%) had not yet heard of DTC‐GT for disease purposes. Therefore, responses were likely influenced by the information provided to the participants before starting the survey (Text S1). In an attempt to reduce intuitive responses, we chose to inform respondents about the different types of genetic tests mentioned in this questionnaire, including those for disease purposes. Explanations were included in the form of written text, short videos, and links to webpages. Some of these videos and webpages were from DTC‐GT companies themselves, which may have given respondents an overly positive view of testing, possibly resulting in more positive responses. However, these were chosen as they provide a good overview of the purpose of each respective test and to reflect easily accessible information (YouTube videos and webpages) which are also likely to be consulted by the general public in real life. (3) Selection bias may have occurred due to the exclusion of incomplete questionnaires. Non‐respondents who did not submit the questionnaire may have been less interested in the topic DTC‐GT compared to respondents. This could potentially explain the high percentage of respondents reporting a genetic disease in the family (22.3%), as these individuals may have had a stronger interest in DTC‐GT. The exclusion of individuals potentially less interested in the topic may have skewed the results toward a more positive outlook. Conversely, the high percentage of respondents reporting a genetic disease in the family may also reflect a broad interpretation of this question. It is possible that respondents were uncertain about what constitutes a genetic disorder, indicating a limitation in the clarity of the question. This is supported by the high proportion of “don't know” responses to this question in both years (2017: 23.4% and 2022: 20.1%). (4) Overlap between participants in the current survey and the survey conducted in 2017 cannot be ruled out. However, Flycatcher panel members cannot select the types of surveys they wish to participate in, ensuring a diverse and representative pool of participants for each survey. Coupled with Flycatcher's extensive and dynamic database of panelists, as well as the 5‐year gap since the previous survey, this suggests that any overlap is likely minimal and unlikely to significantly impact the validity of our findings. 5) Lastly, although it would have been valuable to study DTC carrier testing and disease risk tests separately, these tests are often offered in the same DTC‐GT package, and therefore studying them together reflects the real‐life situation.

## 5 CONCLUSION

This survey indicates that more people are forming an opinion about testing compared to five years ago. Overall, acceptability of DTC‐GT for disease‐related purposes is still low in the Netherlands, while at the same time, consideration is up from 1/8 in 2017 to 1/3 in 2022. Although less pronounced, there is also a shift toward increased intention to take a DTC‐GT for disease‐related purposes within the next year. The characteristics identified in this study can provide valuable insights to inform the development and distribution of public information materials and guidance around DTC‐GT. Genetic counselors could offer important and unique expertise in the development of such materials. Future research should focus on (1) prospective studies on actual uptake of DTC‐GT in the Netherlands and associated factors, (2) the development of freely accessible information materials on DTC‐GT, and (3) identifying the best way to direct people toward reliable DTC‐GT information.

## AUTHOR CONTRIBUTIONS

DK designed the original questionnaire, which was adapted by ARL in collaboration with AW and MZ. The data were collected by the internet research company Flycatcher (Maastricht, The Netherlands) (Flycatcher Internet Research, 2024). All data analyses and the writing of the first draft of the manuscript were carried out by ARL. AW, DK, and MZ critically revised the manuscript for important intellectual content. All authors approved the final manuscript to be published and agree to be accountable for all aspects of the work in ensuring that questions related to the accuracy or integrity of any part of the work are appropriately investigated and resolved.

## FUNDING INFORMATION

The authors declare that no funds, grants, or other support were received during the preparation of this manuscript.

## CONFLICT OF INTEREST STATEMENT

Anna Roos Leerschool, Anke Wesselius, Daša Kokole, and Maurice P. Zeegers declare that they have no conflict of interest.

## ETHICS STATEMENT

Human studies and informed consent: This study protocol was reviewed and approved by the Ethics Review Committee of Maastricht University (FHML‐REC/2021/085). All persons gave online informed consent prior to their inclusion in the study.

Animal studies: No non‐human animal studies were carried out for this study.

## Supporting information


Table S1

Data S1

## Data Availability

The data that support the findings of this study are available from the corresponding author upon reasonable request.

## References

[jgc41919-bib-0001] Ajzen, I. (1991). The theory of planned behavior. Organizational Behavior and Human Decision Processes, 50(2), 179–211. 10.1016/0749-5978(91)90020-T

[jgc41919-bib-0003] Armitage, C. J. , & Conner, M. (2001). Efficacy of the theory of planned behaviour: A meta‐analytic review. British Journal of Social Psychology, 40(4), 471–499. 10.1348/014466601164939 11795063

[jgc41919-bib-0004] Bemelmans, W. , Engelaar, M. , Holst, L. , & Bos, N. (2023). Meningen en emoties van burgers rond DNA‐diagnostiek in zorg en preventie. Nivel.

[jgc41919-bib-0005] Bloss, C. S. , Ornowski, L. , Silver, E. , Cargill, M. , Vanier, V. , Schork, N. J. , & Topol, E. J. (2010). Consumer perceptions of directto‐consumer personalized genomic risk assessments. Genetics in Medicine, 12(9), 556–566. 10.1097/GIM.0b013e3181eb51c6 20717041

[jgc41919-bib-0006] Blout Zawatsky, C. L. , Bick, D. , Bier, L. , Funke, B. , Lebo, M. , Lewis, K. L. , Orlova, E. , Qian, E. , Ryan, L. , Schwartz, M. L. B. , & Soper, E. R. (2023). Elective genomic testing: Practice resource of the National Society of Genetic Counselors. Journal of Genetic Counseling, 32(2), 281–299. 10.1002/jgc4.1654 36597794

[jgc41919-bib-0007] Bos, I. , de Jong, J. , & Verheij, R. (2022). Health checks en de gevolgen voor zorggebruik in 2021. Nivel.

[jgc41919-bib-0008] Calabrò, G. E. , Sassano, M. , Tognetto, A. , & Boccia, S. (2020). Citizens' attitudes, knowledge, and educational needs in the field of omics sciences: A systematic literature review. Frontiers in Genetics, 11, 570649. 10.3389/fgene.2020.570649 33193671 PMC7644959

[jgc41919-bib-0009] Carroll, N. M. , Blum‐Barnett, E. , Madrid, S. D. , Jonas, C. , Janes, K. , Alvarado, M. , Bedoy, R. , Paolino, V. , Aziz, N. , McGlynn, E. A. , & Burnett‐Hartman, A. N. (2020). Demographic differences in the utilization of clinical and direct‐to‐consumer genetic testing. Journal of Genetic Counseling, 29(4), 634–643. 10.1002/jgc4.1193 31749259

[jgc41919-bib-0010] Cernat, A. , Bashir, N. S. , & Ungar, W. J. (2022). Considerations for developing regulations for direct‐to‐consumer genetic testing: A scoping review using the 3‐I framework. Journal of Community Genetics, 13(2), 155–170. 10.1007/s12687-022-00582-3 35171498 PMC8941003

[jgc41919-bib-0011] Charbonneau, J. , Nicol, D. , Chalmers, D. , Kato, K. , Yamamoto, N. , Walshe, J. , & Critchley, C. (2020). Public reactions to direct‐to‐consumer genetic health tests: A comparison across the US, UK, Japan and Australia. European Journal of Human Genetics, 28(3), 339–348. 10.1038/s41431-019-0529-8 31645768 PMC7029038

[jgc41919-bib-0012] Cherkas, L. F. , Harris, J. M. , Levinson, E. , Spector, T. D. , & Prainsack, B. (2010). A survey of UK public interest in internet‐based personal genome testing. PLoS One, 5(10), e13473. 10.1371/journal.pone.0013473 20976053 PMC2957412

[jgc41919-bib-0013] Chung, M. W. H. , & Ng, J. C. F. (2016). Personal utility is inherent to direct‐to‐consumer genomic testing. Journal of Medical Ethics, 42(10), 649–652. 10.1136/medethics-2015-103057 27250638

[jgc41919-bib-0014] Critchley, C. , Nicol, D. , Otlowski, M. , & Chalmers, D. (2015). Public reaction to direct‐to‐consumer online genetic tests: Comparing attitudes, trust and intentions across commercial and conventional providers. Public Understanding of Science, 24(6), 731–750. 10.1177/0963662513519937 24553439

[jgc41919-bib-0015] Data and Insights Network . (2024). Gouden Standaard. https://datainsightsnetwork.nl/advies‐en‐tools/gouden‐standaard/

[jgc41919-bib-0016] Dong, X. , Huang, J. , Yi, Y. , Zhang, L. , Li, T. , & Chen, Y. (2022). Factors associated with the uptake of genetic testing for cancer risks: a pathway analysis using the health information national trends survey data. Life (Basel), 12(12), 2024. 10.3390/life12122024 36556389 PMC9786267

[jgc41919-bib-0017] Flycatcher Internet Research . (2024). Onderzoeksbureau Flycatcher. https://www.flycatcher.eu/nl/

[jgc41919-bib-0018] Gerdes, A. M. , Nicolaisen, L. , Husum, E. , Andersen, J. B. , Gantzhorn, M. D. , Roos, L. , & Diness, B. R. (2021). Direct to consumer genetic testing in Denmark—Public knowledge, use, and attitudes. European Journal of Human Genetics, 29(5), 851–860. 10.1038/s41431-021-00810-3 33649540 PMC8110758

[jgc41919-bib-0019] Godin, G. , & Conner, M. (2008). Intention‐behavior relationship based on epidemiologic indices: An application to physical activity. American Journal of Health Promotion, 22(3), 180–182. 10.4278/ajhp.22.3.180 18251118

[jgc41919-bib-0020] Hall, T. O. , Renz, A. D. , Snapinn, K. W. , Bowen, D. J. , & Edwards, K. L. (2012). Awareness and uptake of direct‐to‐consumer genetic testing among cancer cases, their relatives, and controls: The Northwest Cancer Genetics Network. Genetic Testing and Molecular Biomarkers, 16(7), 744–748. 10.1089/gtmb.2011.0235 22731649 PMC4077008

[jgc41919-bib-0021] Harris, A. , Kelly, S. E. , & Wyatt, S. (2013). Counseling customers: Emerging roles for genetic counselors in the direct‐to‐consumer genetic testing market. Journal of Genetic Counseling, 22(2), 277–288. 10.1007/s10897-012-9548-0 23093333 PMC3597267

[jgc41919-bib-0022] Hoxhaj, I. , Stojanovic, J. , & Boccia, S. (2020). European citizens' perspectives on direct‐to‐consumer genetic testing: An updated systematic review. European Journal of Public Health, 33(5), 947–953. 10.1093/eurpub/ckz246 32361734 PMC11227739

[jgc41919-bib-0023] Hsieh, V. , Braid, T. , Gordon, E. , & Hercher, L. (2021). Direct‐to‐consumer genetic testing companies tell their customers to ‘see a genetic counselor’. How do genetic counselors feel about direct‐to‐consumer genetic testing? Journal of Genetic Counseling, 30(1), 191–197. 10.1002/jgc4.1310 32706156

[jgc41919-bib-0002] Hudson, K. , Javitt, G. , Burke, W. , & Byers, P. (2007). ASHG statement on direct‐to‐consumer genetic testing in the United States. The American Journal of Human Genetics, 81(3), 635–637. 10.1097/01.AOG.0000292086.98514.8b 18055737

[jgc41919-bib-0024] Hui, V. C. C. , Li, H. C. , Chow, J. H. K. , Ng, C. S. C. , Lui, C. Y. W. , Fung, J. L. F. , Mak, C. C. Y. , Chung, B. H. Y. , & Lau, K. K. (2021). Understanding and perception of direct‐to‐consumer genetic testing in Hong Kong. Journal of Genetic Counseling, 30(6), 1640–1648. 10.1002/jgc4.1430 33938075

[jgc41919-bib-0025] International Organization for Standardization . (2019). Market, opinion and social research including insights and data analytics (ISO Standard No. 20252:2019). https://www.iso.org/standard/73671.html

[jgc41919-bib-0026] Janzen, T. (2014). Autosomal DNA testing comparison chart. International Society of Genetic Genealogy Wiki. https://isogg.org/wiki/Autosomal_DNA_testing_comparison_chart

[jgc41919-bib-0027] Khan, R. , & Mittelman, D. (2018). Consumer genomics will change your life, whether you get tested or not. Genome Biology, 19(1), 120. 10.1186/s13059-018-1506-1 30124172 PMC6100720

[jgc41919-bib-0028] Mählmann, L. , Röcke, C. , Brand, A. , Hafen, E. , & Vayena, E. (2016). Attitudes towards personal genomics among older Swiss adults: An exploratory study. Applied & Translational Genomics, 8, 9–15. 10.1016/j.atg.2016.01.009 27047754 PMC4796807

[jgc41919-bib-0029] Martins, M. F. , Murry, L. T. , Telford, L. , & Moriarty, F. (2022). Direct‐to‐consumer genetic testing: An updated systematic review of healthcare professionals' knowledge and views, and ethical and legal concerns. European Journal of Human Genetics, 30(12), 1331–1343. 10.1038/s41431-022-01205-8 36220915 PMC9553629

[jgc41919-bib-0030] Mavroidopoulou, V. , Xera, E. , & Mollaki, V. (2015). Awareness, attitudes and perspectives of direct‐to‐consumer genetic testing in Greece: A survey of potential consumers. Journal of Human Genetics, 60(9), 515–523. 10.1038/jhg.2015.58 26040209

[jgc41919-bib-0031] Metcalfe, S. A. , Hickerton, C. , Savard, J. , Stackpoole, E. , Tytherleigh, R. , Tutty, E. , Terrill, B. , Turbitt, E. , Gray, K. , Middleton, A. , Wilson, B. , Newson, A. J. , & Gaff, C. (2019). Australians' perspectives on support around use of personal genomic testing: Findings from the Genioz study. European Journal of Medical Genetics, 62(5), 290–299. 10.1016/j.ejmg.2018.11.002 30439534

[jgc41919-bib-0032] Miura, M. S. , Suckiel, S. A. , Naik, H. , Soper, E. R. , & Abul‐Husn, N. S. (2023). Elective genetic testing: Genetics professionals' perspectives and practices. Journal of Genetic Counseling, 32(3), 607–617. 10.1002/jgc4.1666 36575824

[jgc41919-bib-0033] Morren, M. , Rijken, M. , Baanders, A. N. , & Bensing, J. (2007). Perceived genetic knowledge, attitudes towards genetic testing, and the relationship between these among patients with a chronic disease. Patient Education and Counseling, 65(2), 197–204. 10.1016/j.pec.2006.07.005 16939709

[jgc41919-bib-0034] Mukherjee, M. , Eby, M. , Wang, S. , Lara‐Millán, A. , & Earle, A. M. (2022). Medicalizing risk: How experts and consumers manage uncertainty in genetic health testing. PLoS One, 17(8), e0270430. 10.1371/journal.pone.0270430 35925961 PMC9352100

[jgc41919-bib-0056] Naithani, N. , Sinha, S. , Misra, P. , Vasudevan, B. , & Sahu, R. (2021). Precision medicine: Concept and tools. Medical Journal, Armed Forces India, 77(3), 249–257. 10.1016/j.mjafi.2021.06.021 34305276 PMC8282508

[jgc41919-bib-0036] National Society of Genetic Counselors . (2019). At‐home genetic testing position statement. www.nsgc.org

[jgc41919-bib-0037] Nelson, S. C. , Bowen, D. J. , & Fullerton, S. M. (2019). Third‐party genetic interpretation tools: A mixed‐methods study of consumer motivation and behavior. The American Journal of Human Genetics, 105(1), 122–131. 10.1016/j.ajhg.2019.05.014 31204012 PMC6612532

[jgc41919-bib-0038] Orbell, S. , & Sheeran, P. (1998). ‘Inclined abstainers’: A problem for predicting health‐related behaviour. British Journal of Social Psychology, 37(2), 151–165. 10.1111/j.2044-8309.1998.tb01162.x 9639861

[jgc41919-bib-0039] Pavarini, G. , Hamdi, L. , Lorimer, J. , & Singh, I. (2021). Young people's moral attitudes and motivations towards direct‐to‐consumer genetic testing for inherited risk of Alzheimer disease. European Journal of Medical Genetics, 64(6), 104180. 10.1016/j.ejmg.2021.104180 33781925 PMC8192412

[jgc41919-bib-0040] Rafiq, M. , Ianuale, C. , Ricciardi, W. , & Boccia, S. (2015). Direct‐to‐consumer genetic testing: A systematic review of European guidelines, recommendations, and position statements. Genetic Testing and Molecular Biomarkers, 19(10), 535–547. 10.1089/gtmb.2015.0051 26313927

[jgc41919-bib-0041] Rhodes, R. E. , & De Bruijn, G. J. (2013). How big is the physical activity intention‐behaviour gap? A meta‐analysis using the action control framework. British Journal of Health Psychology, 18(2), 296–309. 10.1111/bjhp.12032 23480428

[jgc41919-bib-0042] Rigter, T. , Jansen, M. E. , van Klink‐de Kruijff, I. E. , & Onstwedder, S. M. (2020). Kansen en risico's van DNA‐zelftesten. Rijksinstituut voor Volksgezondheid en Milieu.

[jgc41919-bib-0051] Rijksinstituut voor Volksgezondheid en Milieu . (2024). Testen op lichaamsmateriaal. https://www.rivm.nl/gezondheidstesten/lichaamsmateriaal

[jgc41919-bib-0043] Roberts, J. S. , Gornick, M. C. , Carere, D. A. , Uhlmann, W. R. , Ruffin, M. T. , & Green, R. C. (2017). Direct‐to‐consumer genetic testing: User motivations, decision making, and perceived utility of results. Public Health Genomics, 20(1), 36–45. 10.1159/000455006 28068660 PMC12834086

[jgc41919-bib-0044] Rogers, E. M. (2003). Diffusion of innovations (5th ed.). Free Press.

[jgc41919-bib-0045] Rose, N. (2001). The politics of life itself. Theory, Culture and Society, 18(6), 1–30. 10.1177/02632760122052020

[jgc41919-bib-0046] Ruhl, G. L. , Hazel, J. W. , Clayton, E. W. , & Malin, B. A. (2019). Public attitudes toward direct to consumer genetic testing. American Medical Informatics Association Annual Symposium Proceedings Archive, 2019, 774–783. PMC7153088 32308873

[jgc41919-bib-0047] Sanderson, S. C. , O'Neill, S. C. , Bastian, L. A. , Bepler, G. , & McBride, C. M. (2010). What can interest tell us about uptake of genetic testing? Intention and behavior amongst smokers related to patients with lung cancer. Public Health Genomics, 13(2), 116–124. 10.1159/000226595 19556750 PMC3696369

[jgc41919-bib-0048] Sheeran, P. (2002). Intention—Behavior relations: A conceptual and empirical review. European Review of Social Psychology, 12(1), 1–36. 10.1080/14792772143000003

[jgc41919-bib-0049] Sheeran, P. , & Webb, T. L. (2016). The intention–behavior gap. Social and Personality Psychology Compass, 10(9), 503–518. 10.1111/spc3.12265

[jgc41919-bib-0050] Stewart, K. F. J. , Kokole, D. , Wesselius, A. , Schols, A. M. W. J. , Zeegers, M. P. , de Vries, H. , & Van Osch, L. A. D. M. (2018). Factors associated with acceptability, consideration and intention of uptake of direct‐to‐consumer genetic testing: A survey study. Public Health Genomics, 21(1‐2), 45–52. 10.1159/000492960 30359983 PMC6425853

[jgc41919-bib-0052] Tiner, J. C. , Mechanic, L. E. , Gallicchio, L. , Gillanders, E. M. , & Helzlsouer, K. J. (2022). Awareness and use of genetic testing: An analysis of the Health Information National Trends Survey 2020. Genetics in Medicine, 24(12), 2526–2534. 10.1016/j.gim.2022.08.023 36136089 PMC9746668

[jgc41919-bib-0053] Vayena, E. (2015). Direct‐to‐consumer genomics on the scales of autonomy. Journal of Medical Ethics, 41(4), 310–314. 10.1136/medethics-2014-102026 24797610 PMC4392219

[jgc41919-bib-0054] Vermeulen, E. , Henneman, L. , van El, C. G. , & Cornel, M. C. (2014). Public attitudes towards preventive genomics and personal interest in genetic testing to prevent disease: A survey study. European Journal of Public Health, 24(5), 768–775. 10.1093/eurpub/ckt143 24068545

[jgc41919-bib-0055] Wasson, K. , Sanders, T. N. , Hogan, N. S. , Cherny, S. , & Helzlsouer, K. J. (2013). Primary care patients' views and decisions about, experience of and reactions to direct‐to‐consumer genetic testing: A longitudinal study. Journal of Community Genetics, 4(4), 495–505. 10.1007/s12687-013-0156-y 23832288 PMC3773312

