# Supplementary material for: Changes in acceptability, consideration, intention, and uptake of direct‐to‐consumer genetic tests in the Netherlands from 2017 to 2022
Source: J Genet Couns. 2024 Jun 3;34(1):e1919. doi: 10.1002/jgc4.1919 (PMC11735180; doi:10.1002/jgc4.1919)
Supplement: Supplementary file 1 — Table S1 [file JGC4-34-0-s001.docx]

**Supplementary Table 1. Univariable analyses for acceptability, consideration and intention of DTC-GT for disease-related purposes**

| Acceptability |  | **b** | **SE b** | **p-value** | **Non- linearity test (p)#** |
| --- | --- | --- | --- | --- | --- |
| Gender | Female | -0.190 | 0.120 | 0.113^$^ | N/A |
|  | Male | Ref |  |  |  |
| Age in years | Per 1 year increase | -0.005 | 0.004 | 0.171^$^ | 0.004* |
| Age in categories | 18-24 | Ref |  |  | N/A |
|  | 25-34 | 0.120 | 0.252 | 0.633 |  |
|  | 35-44 | 0.302 | 0.249 | 0.225 |  |
|  | 45-54 | 0.167 | 0.248 | 0.501 |  |
|  | 55-64 | 0.137 | 0.247 | 0.579 |  |
|  | 65-74 | 0.032 | 0.251 | 0.900 |  |
|  | 75+ | -0.538 | 0.309 | 0.082^$^ |  |
| Education | Less | Ref |  |  | N/A |
|  | Medium | -0.031 | 0.151 | 0.836 |  |
|  | High | -0.428 | 0.161 | 0.008* |  |
| Having a partner | Yes | 0.176 | 0.132 | 0.183^$^ | N/A |
|  | No | Ref |  |  |  |
| Being religious | Yes | -0.061 | 0.127 | 0.630 | N/A |
|  | No | Ref |  |  |  |
| Planning to have children | Yes | -0.184 | 0.160 | 0.250 | N/A |
|  | Maybe | 0.510 | 0.280 | 0.069^$^ |  |
|  | Don’t know | 0.733 | 0.308 | 0.017* |  |
|  | No | Ref |  |  |  |
| Having biological children | Yes | -0.112 | 0.122 | 0.355 | N/A |
|  | No | Ref |  |  |  |
| Having adopted or step children | Yes | 0.166 | 0.189 | 0.381 | N/A |
|  | No | Ref |  |  |  |
| Genetic disease in the family | Yes | -0.034 | 0.150 | 0.820 | N/A |
|  | I would rather not say/  don’t know | 0.185 | 0.155 | 0.230 |  |
|  | No | Ref |  |  |  |
| Having a chronic disease | Yes | -0.336 | 0.127 | 0.008* | N/A |
|  | I would rather not say/  don’t know | -0.523 | 0.330 | 0.113^$^ |  |
|  | No | Ref |  |  |  |
| Self-rated health | Per 1 point increase in score | 0.035 | 0.068 | 0.603 | 0.360 |
| Consideration |  | **b** | **SE b** | **p-value** | **Non- linearity test (p)#** |
| Gender | Female | 0.037 | 0.118 | 0.756 | N/A |
|  | Male | Ref |  |  |  |
| Age in years | Per 1 year increase | -0.022 | 0.004 | <0.001* | 0.443 |
| Education | Less | Ref |  |  | N/A |
|  | Medium | 0.405 | 0.151 | 0.007* |  |
|  | High | 0.017 | 0.158 | 0.915 |  |
| Having a partner | Yes | 0.212 | 0.130 | 0.103^$^ | N/A |
|  | No | Ref |  |  |  |
| Being religious | Yes | -0.208 | 0.126 | 0.098^$^ | N/A |
|  | No | Ref |  |  |  |
| Planning to have children | Yes | 0.501 | 0.158 | 0.002* | N/A |
|  | Maybe | 0.871 | 0.282 | 0.002* |  |
|  | Don’t know | 0.436 | 0.309 | 0.158^$^ |  |
|  | No | Ref |  |  |  |
| Having biological children | Yes | -0.219 | 0.120 | 0.069^$^ | N/A |
|  | No | Ref |  |  |  |
| Having adopted or step children | Yes | 0.340 | 0.188 | 0.071^$^ | N/A |
|  | No | Ref |  |  |  |
| Genetic disease in the family | Yes | 0.193 | 0.147 | 0.190^$^ | N/A |
|  | I would rather not say/  don’t know | 0.189 | 0.153 | 0.218 |  |
|  | No | Ref |  |  |  |
| Having a chronic disease | Yes | -0.024 | 0.125 | 0.845 | N/A |
|  | I would rather not say/ don’t know | -0.078 | 0.319 | 0.807 |  |
|  | No | Ref |  |  |  |
| Self-rated health | Per 1 point increase in score | 0.009 | 0.067 | 0.898 | 0.548 |
| Intention |  | **b** | **SE b** | **p-value** | **Non- linearity test (p)#** |
| Gender | Female | -0.146 | 0.123 | 0.235 | N/A |
|  | Male | Ref |  |  |  |
| Age in years | Per 1 year increase | -0.003 | 0.004 | 0.456 | 0.109 |
| Education | Less | Ref |  |  | N/A |
|  | Medium | -0.060 | 0.155 | 0.696 |  |
|  | High | -0.321 | 0.165 | 0.052^$^ |  |
| Having a partner | Yes | 0.031 | 0.136 | 0.820 | N/A |
|  | No | Ref |  |  |  |
| Being religious | Yes | 0.032 | 0.130 | 0.805 | N/A |
|  | No | Ref |  |  |  |
| Planning to have children | Yes | 0.090 | 0.163 | 0.580 | N/A |
|  | Maybe | 0.475 | 0.286 | 0.097^$^ |  |
|  | Don’t know | -0.077 | 0.323 | 0.811 |  |
|  | No | Ref |  |  |  |
| Having biological children | Yes | 0.066 | 0.125 | 0.595 | N/A |
|  | No | Ref |  |  |  |
| Having adopted or step children | Yes | 0.190 | 0.194 | 0.327 | N/A |
|  | No | Ref |  |  |  |
| Genetic disease in the family | Yes | 0.151 | 0.153 | 0.325 | N/A |
|  | I would rather not say/ don’t know | 0.179 | 0.159 | 0.260 |  |
|  | No | Ref |  |  |  |
| Having a chronic disease | Yes | -0.010 | 0.130 | 0.937 | N/A |
|  | I would rather not say/ don’t know | 0.348 | 0.326 | 0.286 |  |
|  | No | Ref |  |  |  |
| Self-rated health | Per 1 point increase in score | -0.114 | 0.070 | 0.104^$^ | 0.712 |

Legend: * p<0.05; ^$^ 0.05<p<0.20; # If the non-linearity test was significant at p<0.05, an additional univariable analysis was done with the variable as categorical variable and was entered into the multivariable analyses as a categorical variable.
